# Non-Invasive Detection of Anaemia Using Digital Photographs of the Conjunctiva

**DOI:** 10.1371/journal.pone.0153286

**Published:** 2016-04-12

**Authors:** Shaun Collings, Oliver Thompson, Evan Hirst, Louise Goossens, Anup George, Robert Weinkove

**Affiliations:** 1 Wellington School of Medicine, University of Otago, Wellington, New Zealand; 2 Malaghan Institute of Medical Research, Wellington, New Zealand; 3 Sensing and Automation Group, Callaghan Innovation, Lower Hutt, New Zealand; 4 Photography Department, Capital & Coast District Health Board, Wellington, New Zealand; 5 Wellington Blood & Cancer Centre, Capital & Coast District Health Board, Wellington, New Zealand; 6 Department of Pathology & Molecular Medicine, University of Otago Wellington, Wellington, New Zealand; University of Campinas, BRAZIL

## Abstract

**Background and Aims:**

Anaemia is a major health burden worldwide. Although the finding of conjunctival pallor on clinical examination is associated with anaemia, inter-observer variability is high, and definitive diagnosis of anaemia requires a blood sample. We aimed to detect anaemia by quantifying conjunctival pallor using digital photographs taken with a consumer camera and a popular smartphone. Our goal was to develop a non-invasive screening test for anaemia.

**Patients and Methods:**

The conjunctivae of haemato-oncology in- and outpatients were photographed in ambient lighting using a digital camera (Panasonic DMC-LX5), and the internal rear-facing camera of a smartphone (Apple iPhone 5S) alongside an in-frame calibration card. Following image calibration, conjunctival erythema index (EI) was calculated and correlated with laboratory-measured haemoglobin concentration. Three clinicians independently evaluated each image for conjunctival pallor.

**Results:**

Conjunctival EI was reproducible between images (average coefficient of variation 2.96%). EI of the palpebral conjunctiva correlated more strongly with haemoglobin concentration than that of the forniceal conjunctiva. Using the compact camera, palpebral conjunctival EI had a sensitivity of 93% and 57% and specificity of 78% and 83% for detection of anaemia (haemoglobin < 110 g/L) in training and internal validation sets, respectively. Similar results were found using the iPhone camera, though the EI cut-off value differed. Conjunctival EI analysis compared favourably with clinician assessment, with a higher positive likelihood ratio for prediction of anaemia.

**Conclusions:**

Erythema index of the palpebral conjunctiva calculated from images taken with a compact camera or mobile phone correlates with haemoglobin and compares favourably to clinician assessment for prediction of anaemia. If confirmed in further series, this technique may be useful for the non-invasive screening for anaemia.

## Introduction

Anaemia is defined as a quantitative reduction of haemoglobin, the oxygen-carrying component of red blood cells [1]_._ The World Health Organisation (WHO) estimated that 24.8% of the global population was anaemic between 1993 and 2005 [2]. The gold standard for anaemia diagnosis is *ex-vivo* measurement of haemoglobin concentration in whole blood. This method requires venepuncture and specialised equipment, which may introduce delays or be unavailable in resource-poor settings [3]. Point of care testing methods for anaemia are widely available and typically involve analysis of blood by finger-prick sample. These methods are rapid and inexpensive but require liquid reagents and may expose healthcare workers to risks of blood-borne infections [4].

To rapidly screen for anaemia, clinicians often examine for conjunctival pallor. This involves subjective evaluation of the colour of the conjunctival membrane, with the presence of pallor indicating anaemia. Although this clinical sign can be useful, conjunctival pallor has a low sensitivity and specificity for prediction of anaemia and inter-observer agreement is poor [5–7]. Assessment can be improved by the use of colour-scale cards, which are compared directly to the conjunctiva, resulting in improved inter-observer agreement, sensitivity and specificity [8].

Haemoglobin predominantly absorbs green light and reflects red light, and as a result haemoglobin concentration affects tissue colour [9]. An “erythema index” (EI) has been developed to objectively quantify the degree of erythema of skin lesions, using digital photography followed by analysis of the red and green components of images [9,10].

In this study, we aimed to determine whether a conjunctival EI calculated from digital photographs taken in ambient lighting conditions correlates with haemoglobin concentration. Our goal was to develop a non-invasive method of anaemia detection using a consumer camera or smartphone. We found that EI of the palpebral conjunctiva correlated with haemoglobin concentration and compared favourably with clinician assessment. Our findings suggest that quantification of conjunctival pallor using a digital camera or smartphone has potential application as a non-invasive and affordable screening method for anaemia.

## Methods

### Study design

This was a cross-sectional observational study of hospital inpatients and outpatients attending Wellington Blood and Cancer Centre, a regional haemato-oncology service. Inclusion criteria were: age 18 years or over and laboratory haemoglobin measurement within 36 hours of photography. Exclusion criteria were: active ocular disease, current use of eye drops, total body or ocular radiation within the preceding two weeks, known hypoxia (SpO_2_ < 90%), inability or unwillingness to provide written consent, or participant receiving or due to receive blood transfusion between the time of photography and laboratory haemoglobin measurement. All participants provided written informed consent. The University of Otago Human Research Ethics Committee approved this study (reference HE14/014).

### Conjunctival photography

Conjunctival photographs were taken using two consumer cameras: a Panasonic DMC-LX5 digital camera (LX5; Panasonic Corporation, Osaka, Japan; 3648 x 2736 [10,100,000] pixel, 1/1.63 inch Charge-Coupled Device [CCD], 12-bit sensor bit depth) and the internal rear camera of a popular smartphone (iPhone 5S; Apple Incorporated, CA, USA; 3264 x 2448 [8,000,000] pixel, 1/3 inch Complementary Metal Oxide Semi-Conductor [CMOS], 8-bit sensor bit depth) using the default ‘Camera’ application (iOS version 8.1; Apple Inc.).

Study participants were asked to hold a colour calibration card in one hand (X-Rite ColorChecker® Nano, Edmund Optics, Singapore), and to pull the lower conjunctiva downwards with the other hand. Photographs were taken at a distance of approximately 40 cm, and framed to include the eye and colour calibration card within the same image and the same coronal plane. Automatic focus was used throughout. LX5 photographs were taken without flash and saved using the RW2 file format (the Panasonic ‘raw’ format). The internal white balancing feature was utilised in the LX5, using a white sheet of paper as a reference. iPhone images were taken with automatic white balance, without flash and saved using the Joint Photographic Expert Group (JPEG) file format.

To assess reproducibility, five conjunctival photographs were taken from each of three healthy participants in each of three different lighting conditions using the LX5 camera. For patient participants, three photographs were taken using each of the two cameras in ambient lighting.

### Image analysis

Images were imported and catalogued using a personal computer running Windows 8.1 (Microsoft Corporation, WA, USA). Images were processed using ImageJ version 1.48v, a freeware java-based image software available from the National Institutes of Health website [11]. LX5 RW2 (‘raw’) images were imported into ImageJ using the DCRAW reader plugin (version 1.5.0) [12].

Each image was visually assessed before analysis. The first of the three images meeting all of the following criteria was selected for analysis: conjunctiva and colour card both subjectively in focus; adequate conjunctiva exposed to identify forniceal and palpebral portions and the demarcation between them; no bright conjunctival reflections; no difference in lighting between conjunctiva and colour card. If no suitable image was available for analysis, the participant was excluded.

All images were standardised to enable comparison using a previously established method [13]. First, each image was split into its component 8-bit red, green and blue channels. Each channel’s brightness was adjusted by multiplying its brightness by 200/M_B_ where M_B_ is the mean brightness of the colour calibration card’s white square. At this point, the channels were duplicated, with one set merged to produce a 24-bit white-balanced image. This combined image was then saved in the Tagged Image File Format (TIFF) for future clinician assessment. The other set of the standardised colour channels was then used for EI analysis.

The EI was determined using the equation reported by Yamamoto et al [10]: EI = log(*S*_*red*_) − log(*S*_*green*_) where *S* is the brightness of the conjunctiva in the relevant colour channel. To calculate this, the log function within ImageJ was used on both the red and green channels individually. As these are 8-bit colour channels, the in-built log function scales the result by a fixed factor to allow for results within the pixel scale of 0–255. Following this, we used the Image Calculator tool to subtract the log green channel from the log red channel. In the image produced by this analysis, the intensity of the pixel brightness values is the EI. Palpebral and forniceal portions of the conjunctiva were selected individually using the ‘polygon’ tool to maximise sampling area and measured on the EI image. A macro was written within ImageJ to semi-automate this process, and is provided in the supporting information files (S1 File).

### Clinician Assessment

A slideshow of the standardised LX5 images was produced. Three clinicians were asked to subjectively evaluate each image for conjunctival pallor. Images were displayed on a liquid crystal display colour monitor (Viewsonic VP191s, ViewSonic Europe Ltd, London, United Kingdom), calibrated using a Spyder3Pro® device and software (DataColor Imaging Solutions, Lawrenceville, NJ, USA). Clinicians were first shown three example images of participants with anaemia, and three of non-anaemic participants. They were then shown the images of all study participants in a random order, and asked to rank each as “anaemic” or “not anaemic”. Ten of the participant images were duplicated, flipped horizontally and shown non-consecutively to assess intra-clinician consistency; the first viewing of each image was used to assess clinician detection of anaemia.

### Laboratory haemoglobin measurement

Venous blood was drawn into ethylenediaminetetraacetic acid- containing blood collection tubes (Vacutainer, BD, Auckland, New Zealand). Haemoglobin was measured in an accredited clinical laboratory accredited using a sodium lauryl sulphate haemoglobin detection method (Sysmex XE-2100, Roche Diagnostics, Auckland, New Zealand).

### Statistical analyses

Microsoft Excel 2013 (Microsoft Corporation, WA, USA) and GraphPad Prism (Version 5.0 for Mac OS X, Graphpad Software, CA, USA) were used for statistical analyses. A two-sided *P* value of < 0.05 was considered significant throughout.

## Results

### Study Participants

One hundred and six patient participants were recruited over seven weeks. Of these, five were excluded: three due to a lack of laboratory haemoglobin measurement within 36 hours of photography, and two because of the current use of topical ophthalmic medications. Of the remaining 101 participants, photographs were excluded for technical reasons in seven cases: four due to inadequate visualisation of the conjunctival mucosa and three due to shadows leading to uneven lighting of the colour calibration card and conjunctiva. This left 94 participants available for analysis.

Mean age was 62 years (standard deviation (SD) 14; 95% confidence interval (CI) 59 to 65); 52% were male. 85% of participants were of European ancestry, 7% New Zealand Māori with the remaining 9% a mix of other ethnicities. Mean haemoglobin was 113 g/L (SD 21; 95% CI 109 to 117). Forty percent of participants were anaemic, defined as haemoglobin < 110 g/L. The median delay between photography and blood sampling was 6 hours (range 0–31; interquartile range (IQR) 1–24).

### Effect of image standardisation and reproducibility of images

To assess the effect of image standardisation and reproducibility of the calculated erythema index, five images were taken from each of three healthy individuals, each under three different ambient lighting conditions (fluorescent, incandescent and daylight). Images were analysed, generating EI values both pre- and post-standardisation. To assess the effect of ambient lighting conditions on palpebral conjunctival EI, we conducted a two-way ANOVA on values both pre- and post-standardisation. In the pre-standardisation data, ambient lighting was a larger source of variation than in the post-standardisation EI values (F statistic 161.7 versus 101.5, respectively). Reproducibility between images taken under the same conditions was high: across the nine sets of images (three participants each under three lighting conditions), average coefficient of variation of the conjunctival EI calculated from standardised images was 2.96% (range 1.73–4.55%).

### Associations between palpebral and forniceal conjunctival EI, haemoglobin, and processing time

**Fig 1:**
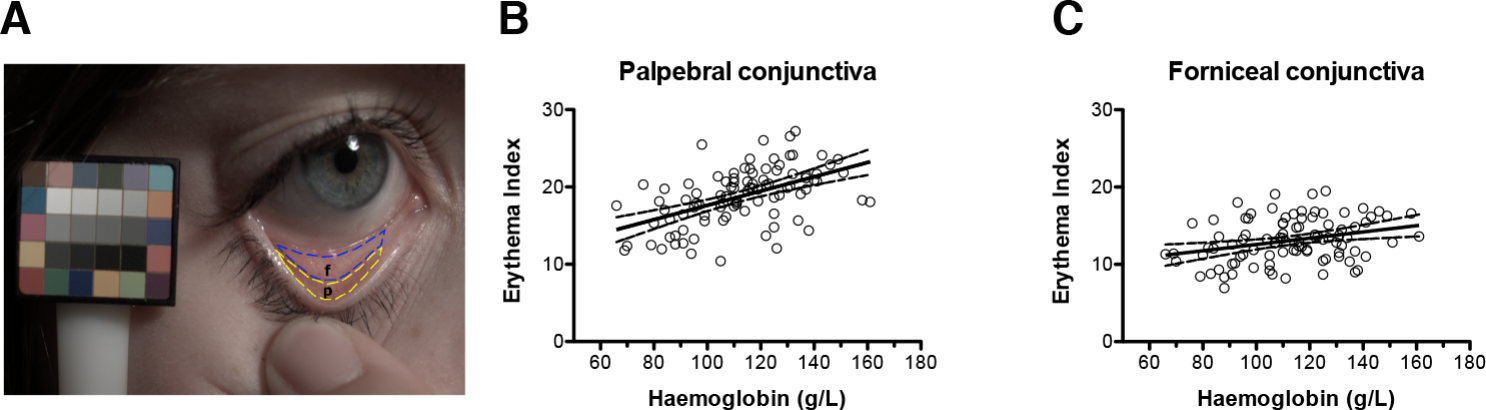
Relationship between erythema index and haemoglobin for palpebral and forniceal conjunctiva. (A) Representative calibrated image from a non-anaemic participant showing the palpebral (p) and forniceal (f) portions of the conjunctiva. The in-frame colour calibration target is shown. (B) Relationship between palpebral conjunctival EI derived from LX5 images and measured haemoglobin (n = 94). Solid line represents best fit by linear regression; slope = 0.092 ± 0.016; *P* < 0.0001 of zero slope. Dashed line represents 95% confidence interval. (C) Relationship between forniceal conjunctival EI derived from LX5 images and measured haemoglobin (n = 94). Solid line represents best fit by linear regression; slope = 0.040 ± 0.014; *P* = 0.004 of zero slope. Dashed line represents 95% confidence interval.

The palpebral portion of the conjunctiva is more erythematous than the forniceal portion (Fig 1A). Digital analysis of the LX5 images confirmed this, with significantly higher EI values for the palpebral than the forniceal portion (paired t test, *P* < 0.0001). As haemoglobin and both palpebral and forniceal conjunctiva EI values followed normal distributions (*P* = 0.60 and *P* = 0.21 respectively, D’Agostino & Pearson omnibus normality test), we performed two separate linear regressions using the sum-of-squares method. We found a significant relationship between palpebral conjunctival EI and measured haemoglobin (r^2^ = 0.27; Fig 1B). A relationship between forniceal EI and haemoglobin concentration was also observed but was weaker (r^2^ = 0.09, Fig 1C). Palpebral conjunctival EI was therefore used for all further analyses. We then proceeded to evaluate the effect of time delay between blood sampling and photography. We split the participants into two groups: those in whom photographs were taken within four hours of blood sample collection (n = 41), and those in whom they were taken over 12 hours before or after blood collection (n = 40). Linear regression using the sum-of-squares method showed similar relationships between palpebral conjunctival EI and haemoglobin in both groups (r^2^ = 0.397 versus r^2^ = 0.307, respectively).

### Sensitivity and specificity of anaemia detection using consumer camera and a smartphone

To assess the diagnostic utility of conjunctival EI analysis, we first had to select an EI threshold that would produce the most clinically useful tool with regards to sensitivity and specificity. First, we allocated alternate patients’ images to training and validation sets (n = 47 for each set). Within the training set, we separated the group into anaemic (n = 15) or not anaemic (n = 32) patients using a haemoglobin cut-off of 110 g/L. We then conducted receiver operator characteristic (ROC) analyses on the training set. The EI value resulting in the greatest sum of sensitivity and specificity was selected as the EI ‘threshold’ for detection of anaemia.

**Fig 2:**
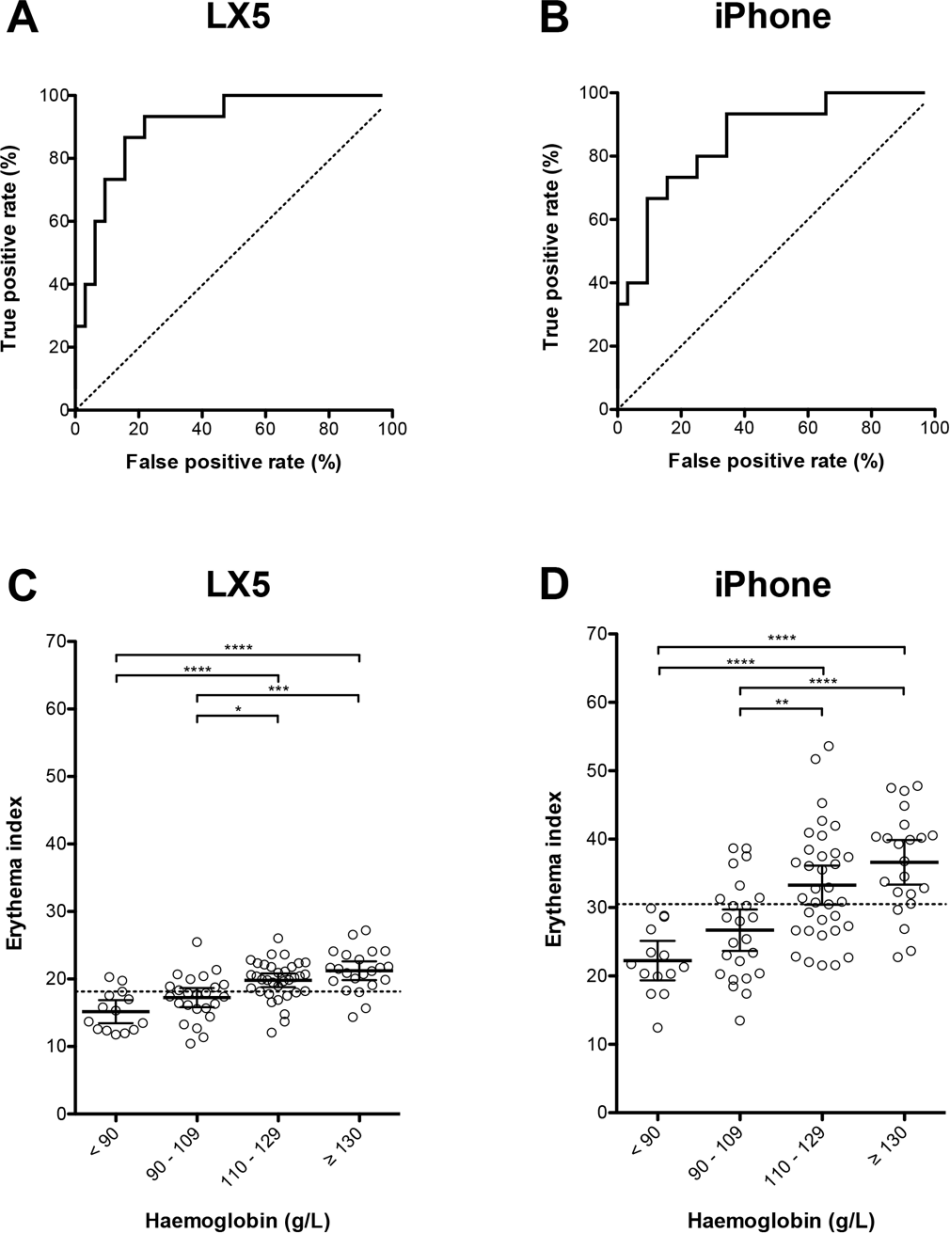
Test characteristics of LX5 and iPhone images. (A) Receiver-operator characteristic (ROC) curve (continuous line) of palpebral conjunctival EI calculated from LX5 camera images for detection of haemoglobin < 110 g/L in the training set (n = 47). Dashed line represents unity. (B) ROC curve (continuous line) of palpebral conjunctival EI calculated from iPhone 5S images for detection of haemoglobin < 110 g/L in the training set (n = 47). Dashed line represents unity. (C) Palpebral conjunctival EI values calculated from LX5 images (n = 94), grouped by haemoglobin concentration. Identical data represented in Fig 1B. Bars represent means. Error bars represent 95% confidence intervals. Dashed line represents EI cut-off for anaemia detection of < 18.14. **** *P* < 0.0001; *** *P* < 0.001; *P < 0.05; One way ANOVA with Bonferroni’s multiple comparison test. (D) Palpebral conjunctival EI values calculated from iPhone 5S images (n = 94), grouped by haemoglobin concentration. Bars represent means. Error bars represent 95% confidence intervals. Dashed line represents EI cut-off of < 30.49 for anaemia detection. **** *P* < 0.0001; ** *P* < 0.01; One way ANOVA with Bonferroni’s multiple comparison test.

Using images from the LX5 camera, area under the ROC curve was 0.90 (95% CI 0.82 to 0.99, *P* < 0.0001), and an EI cut-off of < 18.14 resulted in a sensitivity of 93% (95% CI 68 to 100%) and specificity of 78% (95% CI 60 to 91%) for detection of anaemia within the training set (Fig 2A). Using images from the internal camera of an iPhone 5S smartphone, the area under the ROC curve was 0.86 (95% CI 0.74 to 0.97, *P* < 0.0001), and an EI cut-off of < 30.49 resulted in a sensitivity of 93% (95% CI 68 to 100%) and specificity of 66% (95% CI 47 to 81%) for detection of anaemia within the training set (Fig 2B). Despite different thresholds for prediction of anaemia, EI values derived from LX5 photographs showed a similar relationship with haemoglobin concentration to those taken using the iPhone camera (Fig 2C and 2D).

### Performance of digital image analysis compared with clinician assessment

To compare performance of EI analysis of digital images with clinician assessment, we asked three experienced clinicians to assess each of the 47 validation images as anaemic (n = 23) or not (n = 24), based on their assessment of conjunctival pallor. Intra-observer consistency of evaluation of the ten duplicated images was assessed using Cohen’s Kappa (κ). Clinician one was the most consistent with a good strength of agreement (κ = 0.783, *P* = 0.011), while clinicians two and three showed a fair strength of agreement (κ = 0.400, *P* = 0.114; κ = 0.211, *P* = 0.690 respectively).

Digital EI analysis using the LX5 images correctly identified 70% of images as anaemic or not anaemic, whereas the iPhone images correctly identified 72%. All three clinicians were less accurate than the photographic analysis, scoring 60%, 57% and 64% correctly, respectively. Digital EI analysis resulted in a higher positive likelihood ratio and stronger statistical association between conjunctival pallor and proven anaemia than assessment by the three clinicians (Table 1).

**Table 1:** Comparison of palpebral conjunctival EI with clinician assessment.

	LX5 EI	iPhone 5S EI	Clinician 1	Clinician 2	Clinician 3
**Positive predictive value**	0.76 (0.50–0.93)	0.71 (0.49–0.87)	0.59 (0.42–0.74)	0.62 (0.41–0.80)	0.71 (0.48–0.89)
**Negative predictive value**	0.67 (0.47–0.83)	0.74 (0.52–0.90)	0.63 (0.24–0.91)	0.52 (0.30–0.74)	0.58 (0.37–0.77)
**Positive likelihood ratio**	3.39	2.53	1.16	1.29	2.02
**Sensitivity**	0.57 (0.34–0.77)	0.74 (0.52–0.90)	0.88 (0.70–0.98)	0.62 (0.41–0.80)	0.58 (0.37–0.77)
**Specificity**	0.83 (0.63–0.95)	0.71 (0.49–0.87)	0.24 (0.08–0.47)	0.52 (0.30–0.74)	0.71 (0.48–0.89)
***P***	0.007	0.003	0.437	0.388	0.076

Test characteristics of conjunctival pallor assessment by three clinicians compared with digital EI analysis for detection of anaemia (haemoglobin < 110 g/L) in the validation set (n = 47). Threshold of < 18.14 for anaemia detection used for conjunctival EI values derived from LX5 photographs, and threshold of < 30.49 for iPhone photographs. Values in brackets represent 95% confidence intervals. *P* values are using Fisher’s exact test.

## Discussion

In this study, we used an image standardisation technique and a reported method of quantifying erythema of skin lesions (the erythema index, EI) to quantify conjunctival pallor from digital images [10,13]. In this population of hospital in- and outpatients with a high prevalence of anaemia, we found that EI of the palpebral conjunctiva correlated with haemoglobin more strongly than that of the forniceal conjunctiva, and we identified palpebral conjunctival EI thresholds providing a high sensitivity and specificity for detection of anaemia. We were able to replicate detection of anaemia using the internal camera of a popular smartphone. Using an internal validation set, conjunctival EI compared favourably to clinician assessment of the images.

Strengths of this study include the use of non-standardised ambient lighting in a typical clinical environment, comparison to laboratory-measured haemoglobin and the confirmation of our findings using an internal validation set. The study setting allowed recruitment of a population with a high prevalence of anaemia, increasing the chance of establishing a relationship between conjunctival pallor and haemoglobin levels. We took images using two different cameras: a consumer compact camera and the internal camera of a smartphone. Images were suitable for analysis in the majority of cases (93%). The image standardisation technique applied was a straightforward method, which could be automated. The successful use of images from a smartphone in this study indicates that despite automated image processing, the cameras within popular mobile phones may be capable of anaemia screening. Finally, use of the same images offered an unbiased comparison between performance of the objective EI analysis and subjective clinician assessment.

This study has limitations. Although the method of image standardisation used reduced the effect of ambient lighting on EI, it did not eliminate it. The use of an internal control, such as photography of both conjunctivae and subsequent analysis of intra-patient EI differences, might have allowed us to determine the reproducibility of photography and the image white balancing and analysis techniques [14]. As photographs were taken under various lighting conditions, lighting variability may have weakened the observed association between EI and haemoglobin. The EI threshold for detection of anaemia using the LX5 camera and the smartphone differed. This suggests that the image standardisation method used failed to fully correct a colour bias. It is possible that alternative image standardisation techniques could overcome the effects of lighting or device in part. For example, use of the coloured components of the colour calibration target may allow more accurate image standardisation [15,16].

An alternative explanation for the observed difference in EI threshold between devices is that the two cameras employed different internal image processing algorithms, such as dynamic tone mapping, differentially affecting the results. As camera applications in modern smartphones are designed to produce aesthetically pleasing pictures, they may apply automated image adjustments [17]. Nonlinear level adjustments, or adjustments performed differentially on certain portions of the image, may confound colour calibration and affect the EI threshold for prediction of anaemia. As such adjustments are likely to be hardware- and even software version-specific, updates to camera or smartphone hardware or software could affect the predictive value of EI. Any anaemia detection application should therefore have access to the output of standardised camera hardware prior to image adjustments. The introduction of smartphone models supporting raw image formats and manual control of camera settings may provide a solution [18].

Our study design allowed up to 36 hours between conjunctival photography and laboratory-measured haemoglobin. As red cell transfusion was an exclusion criterion, it is unlikely that haemoglobin concentration changed substantially in the period between blood sampling and photography. Moreover, we could find no significant difference in the relationship between EI and haemoglobin between those who had blood sampled within four or beyond 12 hours from the time of photography.

We had to exclude seven sets of photographs due to poor image quality. These images either had shadows differentially affecting the colour calibration target and the conjunctivae, had an inadequate area of conjunctiva exposed for analysis, or were subjectively out of focus. If conjunctival photographs were taken by a larger number of users, a greater proportion of photographs may need to be rejected. Any automated EI analysis software may need to include features to detect these problems objectively and reject the image.

For practical reasons, we evaluated clinician assessment of conjunctival pallor using digital images rather than patients. We were careful to standardise the images and to calibrate the monitor before clinician assessment. The degree of intra-observer consistency we observed suggested that these measures were effective. However, it is possible that clinicians would perform better in clinical practice, perhaps through use of cues other than conjunctival pallor alone. On the other hand, we showed clinicians a set of ‘training’ images before beginning the assessment, an opportunity that is not available in routine clinical practice.

Prior studies have confirmed the utility of both tongue and conjunctival pallor for the screening of severe anaemia (haemoglobin concentration < 70 g/L), but have had limited success at detection of milder anaemia [6,7]. Others have improved test characteristics by combining a clinical history with the use of a colour-scale while assessing for conjunctival pallor, increasing sensitivity from 74% for pallor alone to 83% when medical history and colour-scale were combined. However sensitivity remained low for milder degrees of anaemia [8]. As the clinical utility of a screening test is reliant on the sensitivity, this limits subjective clinician assessment as a point-of-care test. The sensitivity and specificity of a method for detection of anaemia will depend on participant characteristics, such as the haemoglobin threshold and the proportion of those with severe as opposed to mild anaemia. This complicates direct comparison of the results we present to those previously reported. However, our findings are consistent with those in another conjunctival imaging study using the same haemoglobin threshold, which found a sensitivity and specificity of 69% and 72%, respectively [14].

Non-invasive screening for anaemia could have numerous applications as a point of care test, particularly in resource-poor settings. Indeed, a dedicated smartphone ‘app’ to automate image analysis and to predict risk of severe anaemia could be envisaged [19]. This study suggests that conjunctival imaging has potential for this role. However, before it could be used, refinement of the image standardisation technique to reduce the impact of device and lighting characteristics is required, and our findings should be confirmed in other populations and clinical settings and using other camera technologies.

In conclusion, we report a screening technique for the non-invasive detection of anaemia based on digital analysis of the palpebral conjunctiva in a digital photograph. Using either a compact camera or the internal camera of a popular smartphone, we could detect anaemia at reasonable sensitivity and specificity. Conjunctival EI analysis might offer an improvement over clinical assessment of conjunctival pallor. We conclude that digital photography of the conjunctiva may prove useful for the screening of anaemia.

## Supporting Information

S1 File ImageJ Macro. (DOCX)
