# Supplementary material for: Non-Invasive Detection of Anaemia Using Digital Photographs of the Conjunctiva
Source: PLoS One. 2016 Apr 12;11(4):e0153286. doi: 10.1371/journal.pone.0153286 (PMC4829242; doi:10.1371/journal.pone.0153286)
Supplement: S1 File — (DOCX) [file pone.0153286.s001.docx]

## Supplementary Appendix 1: ImageJ Macro

title = getTitle;

run("Rotate 90 Degrees Right");

run("Select None");

run("Split Channels");

wait(100);

selectImage(title+" (red)");

rename("red");

selectImage(title+" (green)");

rename("green");

selectImage(title+" (blue)");

rename("blue");

//standardisation of images (Zhao)

selectImage("red");

setTool("rectangle");

waitForUser("Select the white square");

getStatistics(area, mean, min, max, std, histogram);

muladjR = 200/mean;

print("Red factor " +muladjR);

run("Select None");

run("Multiply...", "value=muladjR");

selectImage("green");

run("Restore Selection");

getStatistics(area, mean, min, max, std, histogram);

muladjG = 200/mean;

print("Green factor " +muladjG);

run("Select None");

run("Multiply...", "value=muladjG");

selectImage("blue");

run("Restore Selection");

getStatistics(area, mean, min, max, std, histogram);

muladjB = 200/mean;

print("Blue factor " +muladjB);

run("Select None");

run("Multiply...", "value=muladjB");

run("Merge Channels...", "c1=[red] c2=[green] c3=[blue] keep");

selectImage("RGB");

rename("Standardised RGB");

//EI transformation

selectImage("red");

run("Select None");

run("Log");

wait(100);

selectImage("green");

run("Select None");

run("Log");

wait(100);

imageCalculator("Subtract create", "red", "green");

rename("EI");

selectImage("Standardised RGB")

setTool("polygon");

waitForUser("Select the palpebral conjunctiva");

selectImage("EI");

run("Restore Selection");

run("Measure");

run("Close All");
